# WSP from *“Nostoc commune” Vauch.* suppresses gastric cancer migration *via* EGFRVIII signaling

**DOI:** 10.3389/fonc.2022.1012863

**Published:** 2022-12-08

**Authors:** Xiaoxia Chen, Wenqi Bai, Xiangrong Liu, Jiao Zhao, Zhiyuan Li, Jianrong Li, Liping Su, Tao Guan, Ruifang Sun, Xihua Yang, Caixia Lv, Zhixiang Wang, Linjie Hu, Zheng Li, Jinfeng Ma, Huanhu Zhang, Xiaoqing Lu

**Affiliations:** ^1^ Shanxi Province Cancer Hospital/Shanxi Hospital Affiliated to Cancer Hospital, Chinese Academy of Medical Sciences/Cancer Hospital Affiliated to Shanxi Medical University, Taiyuan, China; ^2^ Department of General Surgery Sciences, Shanxi Cancer Hospital, Taiyuan, China; ^3^ Department of Pharmacology, School of Medicine, Southern University of Science and Technology, Shenzhen, China; ^4^ Department of Hematology, Shanxi Cancer Hospital, Taiyuan, China; ^5^ Cancer Biobank, Shanxi Cancer Hospital, Taiyuan, Shanxi, China; ^6^ Laboratory Animal Center, Shanxi Cancer Institute, Taiyuan, China; ^7^ School of Fu Shan, Shanxi University of Chinese Medicine, Jin Zhong, China; ^8^ Department of Breast Surgery, Shanxi Cancer Hospital, Taiyuan, China

**Keywords:** WSP, EGFRVIII, metastasis, PI3K-AKT, gastric cancer

## Abstract

**Introduction:**

A number of evidences have proved that “Nostoc commune” Vauch can improve human immunity and prevent diseases, however, the specific mechanism remains unclear. The biological activity of the main protein component of “*Nostoc commune*” *Vauch* extracellular matrix– a water-stress protein (WSP) still needs to be elucidated.

**Methods:**

In our study, we validated the role of WSP in gastric cancer metastasis at the cellular level, the organoid level and in mouse models, and also studied the role of EGFRVIII and downstream signaling molecules after WSP treatment.

**Results:**

We found that WSP can significantly inhibit the metastasis of gastric cancer cells. Interestingly, we found that the anti-metastasis ability of WSP on gastric cancer was related to membrane protein receptor EGFRVIII, which was realized by inhibiting the downstream EGFRVIII signaling pathway. In terms of mechanism, WSP can inhibit the downstream EGFRVIII signaling pathway Akt-PI3K and further inhibit the secretion of cancer-related metastasis proteins such as MMP2 and MMP9, thus, significantly affecting the metastasis of gastric cancer cells.

**Discussion:**

Given the anticancer properties of WSP, drug developers and manufacturers can further develop protein drugs for cancer patients using protein engineering techniques based on the properties of WSP.

## Introduction

Gastric cancer (GC) is the most common type of cancer and most patients are at an advanced stage at the time of diagnosis (1), with unpromising survival rates. The 5-year survival rate of GC patients is poor because of cancer recurrence due to metastases (2).

Water Stress Protein (WSP), derived from Candida commons (3), commonly known as Geodermis, was found to be the most abundant soluble protein in Geodermis as early as 1994, accounting for 83.6% of the total extracellular matrix protein with molecular mass of 33, 37 and 39 kDa. WSP peptides are produced by gene expression and protein hydrolysis, secreted by cells, accumulated in extracellular polysaccharide sheath, and released after rehydration in dried algae (4). Subsequently, we cloned the wspA gene of *“Nostoc commune” Vauch* and found that the transcription of wspA and superoxide dismutase, as well as the synthesis and secretion of wspA, can be induced by cell drying or UV irradiation, demonstrating that recombinant wspA can bind to pseudodendinin through non-covalent interactions to regulate extracellular matrix function (5). In recent years, a series of studies have been conducted on the anticancer effect of WSP. Experimental results show that it can inhibit the proliferation of colon cancer cells by killing cancer cells, while it has almost no effect on normal intestinal epithelial cells. Mechanistically, WSP induces growth inhibition through G1/S blocking and mediates a caspase-dependent pathway to induce apoptosis (6). In addition, WSP can also mediate autophagy-related signaling pathways to affect colon cancer metastasis (7).

Cells sense and respond to stimuli from the external environment through various cell surface signaling molecules (e.g. receptors and ion channels) (8) and membrane proteins can also be activated by cell-cell contact-induced interactions that phosphorylate membrane proteins. These activations may have different consequences for the cell. In most cases, activated membrane proteins induce intrinsic downstream signaling by phosphorylating downstream kinases (9). The epidermal growth factor receptor (EGFR) has obvious mutations in gastric cancer (10) and EGFRVIII protein expression cannot be detected in normal gastric epithelial cells (11). These studies indicated that EGFRVIII protein plays an important role in the process of gastric cancer. During cancer development, cancer metastasis is closely related to cancer prognosis (12). In recent years, studies indicated that WSP1 activated autophagy through downregulation of the PI3K/AKT/mTOR pathway and epithelial-mesenchymal transition (EMT) of DLD-1 cells was restrained through which the migration was subsequently suppressed significantly (7). The above confirmed that WSP has significant anti-colon cancer activity.

Due to the certain similarity in the mechanism of digestive tract cancers, we investigated the anti-migration ability of WSP in gastric cancer cell lines. In the present study, to clarify the role of WSP in gastric cancer, we studied the expression of EGFRVIII in different gastric cancer cell lines, with special attention to its relationship with the biological activity of WSP. Here, we not only explored the effect of WSP on gastric cancer cell migration but also studied the role of EGFRVIII and downstream signaling molecules after WSP treatment.

## Materials and methods

### Ammonium sulfate precipitation

The dried thallus of *“Nostoc commune” Vauch* was purchased from Shuozhou, Shanxi Province. The *“Nostoc commune” Vauch* was washed with distilled water and an appropriate amount of pre-cooled PBS was added. It was rotated and soaked at 4 °C for 48 h, centrifuged at 12,000 rpm for 25 min, and the supernatant which is the crude extract was stored.

We purified WSP using ammonium sulfate precipitation because of its higher protein protection. According to the volume of the above solution, using the ammonium sulfate concentration saturation table, ammonium sulfate was added to the saturation of 30%. After the complete dissolution, it was allowed at 4 °C for 30 min and centrifuged at 11,000 rpm for 10 min. The supernatant was collected and ammonium sulfate was added to make the saturation reach 60%. After the ammonium sulfate was completely dissolved, it was kept at 4 °C for 30 min and centrifuged at 11,000 rpm for 10 min at 4 °C. The precipitate was collected and dissolved in PBS to obtain a solution, which is WSP. The sediment from 30−60% ammonium sulfate was resuspended in PBS, then dialyzed, and concentrated. Proteins were analyzed with a 10% SDS-PAGE. Any activity of fractions is shown later. Gels were stained using R-250. The protein concentration was determined using the bicinchoninic acid (BCA) assay, and the extracts were filtered and decontaminated *via* 0.22 µm filters and stored at -20°C.

### Preparation of recombinant protein

The recombinant strain *E.coli* BL21 containing the WSP gene was inoculated with LB medium containing kanamycin, shaken for 3 hours, induced by the addition of 0.5 mM IPTG, and the incubation was done for 4 hours before ultrasonication lysis and collection of the bacteriophage. After centrifugation of the fragmented bacterium and equilibration of the nickel material, the supernatant of the bacterium was added to a chromatography column for affinity purification. The target protein was eluted with the equilibrium solution, the dialysis eluate was added to a 10KD ultrafiltration tube, concentrated, and stored at -80 °C. The protein concentration was determined *via* BCA method. The extracts were filtered and decontaminated using 0.22 µm filters and stored at -20°C.

### Cell culture

Human colon cancer cell lines SGC-7901, BGC-823, and MKN-28 were purchased from Shanxi college and cultured in RPMI-1640 medium supplemented with 10%(v/v) FBS and 1%;P/S. Cells were cultured at 37 °C, 5% carbon dioxide and 95% humidity.

### Wound healing assay

The gastric cancer cells SGC-7901, BGC-823, and MKN-28 were seeded in a 12-well plate at 500,000 cells per well. When the cells were confluent to 100%, a sterile yellow pipette tip was used perpendicularly to draw a straight line on the culture plate. The scraped suspended cells were gently rinsed with sterile PBS and replaced with a basal medium containing 2% serum and a basal medium containing different concentrations of WSP and 2% serum. After 48 h, the scratch area was photographed and analyzed with a 10x inverted microscope.

### qPCR

Total RNA was extracted from cells using Trizol reagent. cDNA was synthesized from 500ng RNA with PrimeScript RT Master Mix. Quantitative real-time PCR was performed using 2× SYBR Green PCR Master Mix under the following conditions: 95 °C for 30 s, followed by 40 cycles at 95 °C for 5 s, 64 °C for 34 s, and a melt curve step using a Step One Plus Real-Time PCR System (Applied Biosystems). The expression levels of the target gene for each experiment were normalized to GAPDH. The relative gene expression levels were detected and calculated using the ΔΔCt comparative method. The RT-PCR primers used were as follows:

*MMP9* forward: 5-ACCAAGGATATACCTATTCCT-3

*MMP9* reverse: 5-AGGTATAGTGGGACACATAGTGG-3

*MMP2* forward: 5-ACCAAGGATATAGCCTATTCCT-3

*MMP2* reverse: 5-GGCCAGTACCAGTGTCAGTA-3

*EGFRVIII* forward: 5-GGCTCTGGAGGAAAAGAAAGGTAATT-3

*EGFRVIII* reverse: 5-CCGTCTTCCTCCATCTCATAGC-3

*β-actin* forward: 5-CGTGACATTAAGGAGAAGCTG-3

*β-actin* reverse: 5-CTAGAAGCATTTGCGGTGGAC-3

*AKT* forward: 5-TAGCTAGATCGGCTCGATAGCTAGAGGACTAG-3

*AKT* reverse: 5-ATCGATAGCTATATATATGCTAGATGATC-3

*PI3K* forward: 5-ACTCGATAGCTAGATCGATAGCTATAGCTAG-3

*PI3K* reverse: 5-CTGAGCTAGAGCTAGAGTTGCTAGATCGAGT-3

### siRNA mediated knockdown

Knockdown of EGFRvIII was performed using Lipofectamine 3000 according to the manufacturer’s instructions. The following siRNAs were used: EGFRvIII from Eurofins Scientific; [5´-CUGGAGGAAAAGAAAGGUAAU-3´] (13), The medium was changed 5 h after transfection and 24 h later the colony-forming assay was performed as described below.

### Western blotting

The human colon cancer cell line SGC-7901(1 × 10 (6)) was treated with 0.15 μg/μL WSP1 for 24 and 48 h and 72h. After the treatment, cells were incubated with lysis buffer for 30 min on ice and then centrifuged for 10 min at 13000 rcf. Protein concentrations were measured with Bradford reagent (Bio-Rad, USA), and an equal amount of protein (60 μg) from each sample was separated by 10% SDS-PAGE and then transferred to PVDF membranes. The membranes were blocked using Tris-buffered saline with 0.1% Tween-20 and 5% skimmed milk for 1.5 h at room temperature. The membranes were incubated with primary antibodies and HRP-conjugated secondary antibodies and visualized using chemiluminescence (Bio-Rad, USA). Semi-quantitative measurements of band intensity using pixel intensity were performed using ImageJ (NIH, USA) and expressed in densitometric units normalized to the GAPDH.

### Human organoid culture

Approximately 1 cm of cancer tissue was cut into small fragments and washed in cold chelation buffer until the supernatant was clear. Fragments were subjected to enzymatic digestion by collagenase 1.5 mg/mL (Gibco) and hyaluronidase 20 μg/mL (Sigma) in 10 mL Advanced DMEM F12 (GIBCO) supplemented with antibiotics (Primocin, *In vivo*gen) for 1 h at 37 °C with shaking. Cells were washed twice in Advanced DMEM F12, seeded into Matrigel, and overlayed with a medium containing HEPES, Glutamax, Penicillin, Streptomycin, B27, n-Acetylcysteine, EGF, R-spondin1, Noggin, Wnt, FGF10, Gastrin, TGFβ-inhibitor, and RHOK-inhibitor as above.

### Cancer formation assay *in vivo*

All animal experiments were carried out following the procedures approved by the Institutional Animal Care and Use Committee of the China Institute for Radiation Protection. The review board and ethics committee of the institution specifically approved this study. Twenty-four BALB/c nude mice (five-week-old, female, weighing between 18-21 g) were purchased from the Institute of Shanxi cancer hospital, Chinese Academy of Sciences. The nude mice were maintained under specified-pathogen-free (SPF) conditions in the laboratory animal service center of the China Institute for Radiation Protection. SGC-7901 cells in 0.2 mL of PBS (5 × 106) were injected subcutaneously into the right oxter of each nude mouse. Fourteen days later, solid cancers were apparent in all injected nude mice and the 24 nude mice were randomly divided into two groups (each group with 8 nude mice) with no significant difference in cancer volumes between the groups. Mice in the WSP1 group received an intraperitoneal injection of 30, 60 μg WSP1/g body weight every 3 days (a total of six injections), and the control mice were treated with PBS. The body weight and cancer diameters of nude mice were measured twice a week. Then 28 days later, all mice were sacrificed. The number of liver and lung cancer metastases were documented and analysis by immunohistochemistry (IHC) was performed.

### Immunohistochemistry

Liver metastases from BALB/c nude mice (n=10) were collected, fixed in 10% formaldehyde solution, and embedded in conventional paraffin. Paraffin-embedded tissues were cut into 3-μm serial sections and subjected to IHC analysis following standard procedures. Images were captured under a microscope fitted with a charge-coupled device (CCD) camera. High-power field (×200 times) images were collected, and Image-J was used to semi-quantitatively analyze the positive expression of immunohistochemical staining in the sections.

### Statistical analysis

Data analysis was carried out using Graphpad Prism 5.1 software. Results were expressed as mean ± SD. Differences in means between the control and treatment groups were determined using a two-tailed unpaired Student’s t-test or a One-Way ANOVA followed by Tukey’s multiple comparison tests. Values of less than 0.05 (p < 0.05) and 0.01 (p < 0.01) were considered statistically significant and highly significant, respectively.

## Results

### Extraction and purification of WSP

**Figure 1:**
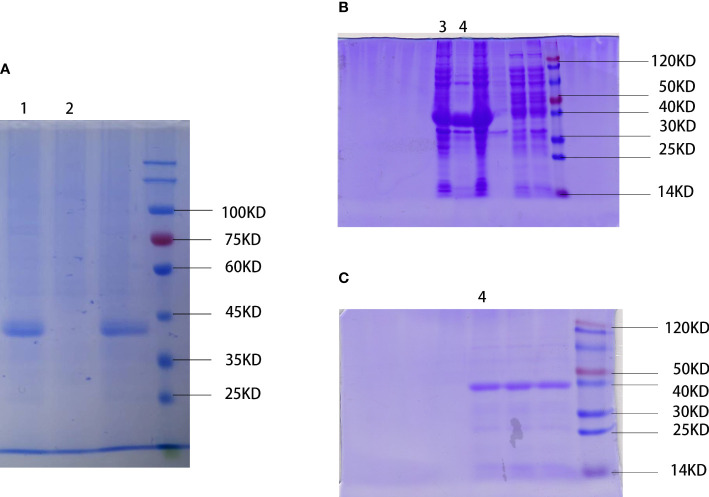
Extraction and preparation of water stress protein. **(A)** Physical extraction of water stress protein using pyruvate precipitation method. WSP is located near the 45 kD of protein marker. 1: WSP of the *“Nostoc commune” Vauch* after ammonium sulfate treatment. 2: crude extract. **(B)** Recombinant water stress protein created by genetic engineering. WSP is located near the 40 kD of protein marker detected by SDS-PAGE. 3: bacteriophage after ultrasonication lysis treatment. 4: WSP after affinity purification. **(C)** Purified water stress protein through nickel column purification (nickel purified), and anion exchange chromatography (anion exchange). 4: WSP after affinity purification.

We physically extracted the WSP, obtained the crude protein extract of “Nostoc commune” Vauch, and condensed the WSP through ammonium sulfate precipitation, as shown in **Figure 1A**. In addition, recombinant proteins produced by genetic engineering technology have a single component, high safety, and controllable production process (24). So, we cloned the single-copy gene of WSP into pET28a through gene recombination technology and mass expression through a prokaryotic system, and obtained WSP1 recombinant protein, as shown in **Figure 1B**. After purification, the purity was up to 90%, as shown in **Figure 1C**.

### The WSP inhibits the migration of gastric cancer cells

**Figure 2:**
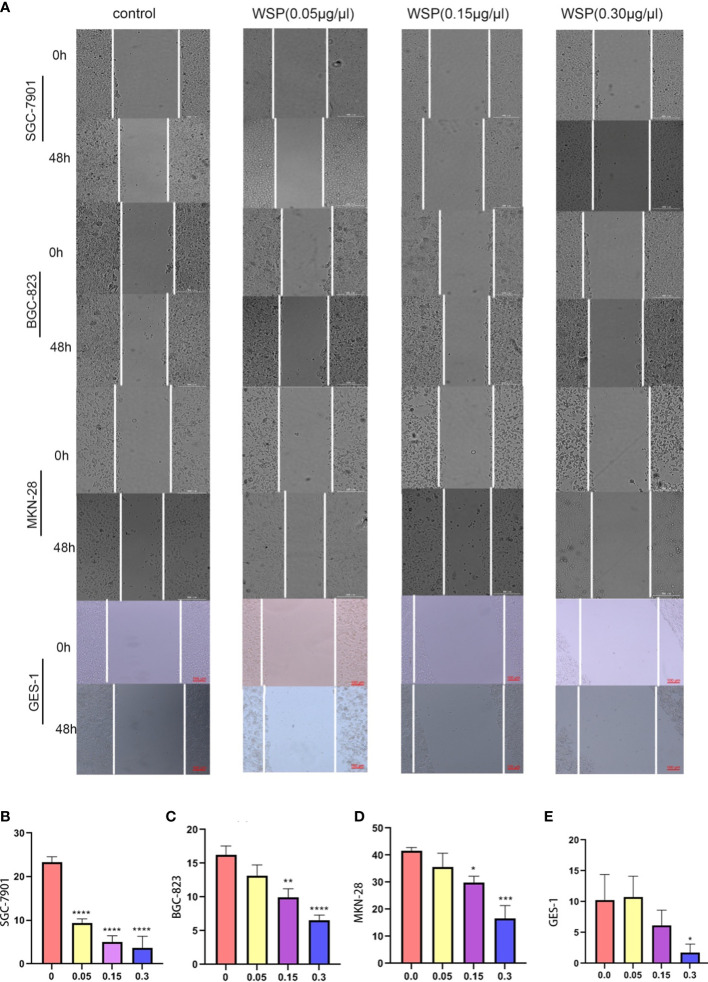
Migration capacity of different cell lines after WSP treatment. **(A)** Migration of SGC-7901, BGC-823, MKN-28, and GES-1 cell lines treated without or with WSP at 0h and 48 h. Bar:300μm. **(B)** Quantification of wound healing percentage of SGC-7901 cells treated without or with WSP after 48h. **(C)** Quantification of wound healing percentage of BGC-823 cells treated without or with WSP after 48h. **(D)** Quantification of wound healing percentage MKN-28 cells, BGC-823, and MKN-28 cells treated without or with WSP after 48h. **(E)** Quantification of wound healing percentage of GES-1 cells treated without or with WSP after 48h. WSP significantly affected the migration of SGC-7901 cells in 0.05μg/μL. *P < 0.05, **P < 0.01, ***P < 0.001, ****P < 0.0001.

In order to explore the effect of WSP on GC migration, SGC-7901, BGC-823, MKN-28, and GES-1 cells were treated with different concentrations of WSP, and their migration ability was compared. The results showed that the migration of gastric cancer cells was significantly inhibited in a dose-dependent manner compared with a control group (**Figure 2A**) Compared with the untreated group, SGC-7901 group showed stronger inhibition of cell migration under different concentrations of WSP treatment than the other two groups (**Figures 2B-D**), but similar to GES-1 (**Figure 2E**). At the same time, it can be seen that under the low dose of WSP treatment, BGC-823 and MKN-28 cell lines have no obvious anti-migration ability compared to the control group, but when the concentration of WSP is 0.3μg/μL, it has a significant effect on the three kinds of cell lines. While GES-1 cells without WSP treatment exhibited no statistically significant differences compared with GES-1 cells with low dose WSP treatment.

In order to further examine the specific effects of WSP on the migration of gastric cancer cells, we used SGC-7901 with relatively significant results for subsequent experiments.

### EGFRVIII is a molecular target of WSP

**Figure 3:**
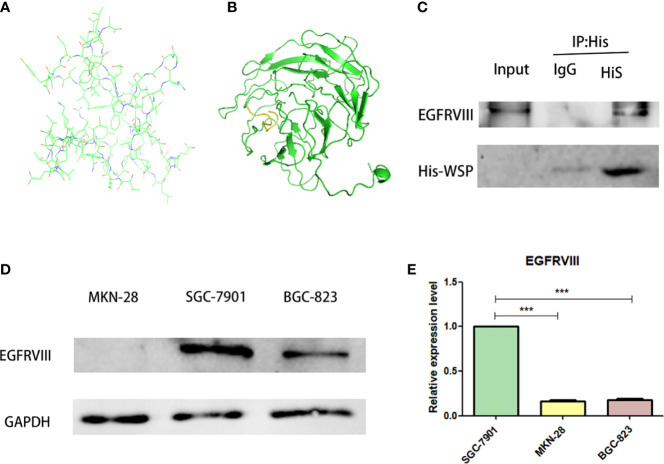
EGFRVIII is a molecular target of WSP. **(A)** WSP 3D protein simulation structure by SWISS-MODEL. **(B)** WSP (the green part) and EGFRVIII protein(the yellow part) simulation binding site by ZDock. **(C)** Immunoprecipitation of EGFRVIII with His-WSP. Sgc-7901 cells were lysed with a potent cell lysate and subjected to immunoprecipitation with isotype control antibody and His label antibody and immunoprecipitated EGFRVIII protein、His-Tag protein were detected with western blot.CO-IP assay to verify WSP binding on EGFRVIII receptor surface. **(D)** Western blotting analysis of EGFRVIII protein expression in SGC-7901, BGC-823, MKN-28 cell lines. **(E)** qPCR analysis of EGFRVIII protein expression in SGC-7901, BGC-823, MKN-28 cell lines. ***P < 0.001.

Based on the known WSP gene fragment, the 3D structure of WSP was predicted in this study. Modeling of protein structure homology has become a conventional technique for simulating protein 3D structure with strong authority (14). Swiss-model is a 3D protein simulation system (15), which is also the most successful tool in protein structure prediction methods (16). Using this tool, we carried out the WSP 3D protein building for its simulation structure (**Figure 3A**). In addition, WSP targets selection is based on the list of priorities of cancer antigens released by the national cancer institute (NCI) (17) from where seven kinds of membrane receptor proteins were selected. The top three proteins were selected to conduct simulated protein-protein docking between the target receptor and WSP. With the ZDOCK tool (18), the corresponding binding site of WSP was found on the EGFRVIII receptor **(** **Figure 3B**). Subsequently, we used a CO-IP assay to confirm the binding of the WSP to the EGFRVIII (**Figure 3C**). Considering that the EGFRVIII target protein expression levels of the three cell lines may lead to the difference in the anti-migration ability of WSPs, we detected the EGFRVIII expression levels of the three cells, and the results showed that the EGFRVIII protein and mRNA expression levels were the highest in SGC-7901 cells (**Figures 3D, E**).

### WSP inhibits PI3K-Akt signaling pathway

**Figure 4:**
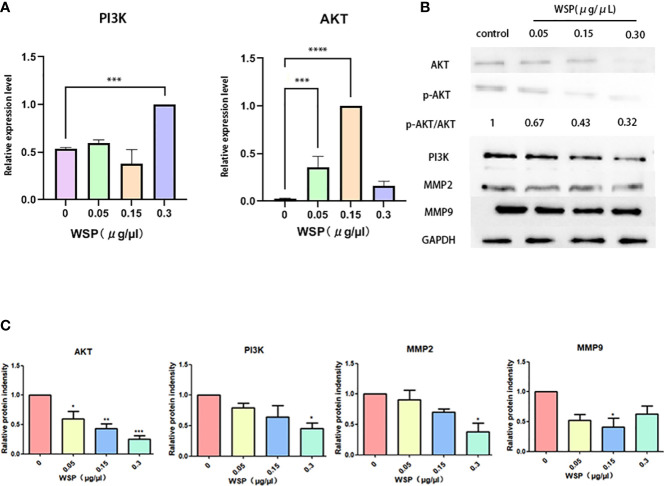
mRNA and protein expressions of SGC-7901 after treatment with different concentrations of WSP. **(A)** qPCR analysis of AKT, PI3K in SGC-7901 treated without or with WSP. **(B)** Western blotting analysis of AKT,p-AKT, PI3K, MMP2, MMP9, and GAPDH in SGC-7901 treated without or with WSP. **(C)** Quantification of AKT, PI3K, MMP2, MMP9 in SGC-7901 cells treated without or with WSP as shown in **(B)**. *P < 0.05, **P < 0.01, ***P < 0.001, ****P < 0.0001.

Since PI3K-Akt is a common downstream signaling pathway of EGFRVIII, we further explored the influence of WSP on PI3K-Akt signaling pathway. Compared with the group without WSP treatment, we detected the mRNA changes of AKT and PI3K at the cellular level, and the results showed that after WSP treatment. In addition, the mRNA expressions of related indicators of cells showed an increased trend (**Figure 4A**). The results demonstrated that transcription was promoted under low-concentration WSP treatment, while transcriptional activity was restored under high-concentration treatment. The significantly decreased levels of AKT, p-AKT, and PI3K expression were observed in SGC-7901 cells in a time- and dose-dependent manner. It demonstrated that the weakening of AKT may be related to the weakening of its phosphorylation level (**Figures 4B, C**). These data demonstrated that WSP treated inhibited p-AKT and PI3K expressions in SGC-7901 cells. Inconsistencies at gene and protein levels are most likely due to post-transcription effects. It can be caused by the modification and degradation of mRNA and the regulation of related miRNAs. Western blot results showed that PI3K-Akt was an important signaling pathway of WSP involved in regulating the migration of SGC-7901. WSP may inhibit the original signaling of membrane protein EGFRVIII, leading to changes in downstream signaling pathways.

### EGFRVIII knockdown reduced the anti-migration ability of WSP

**Figure 5:**
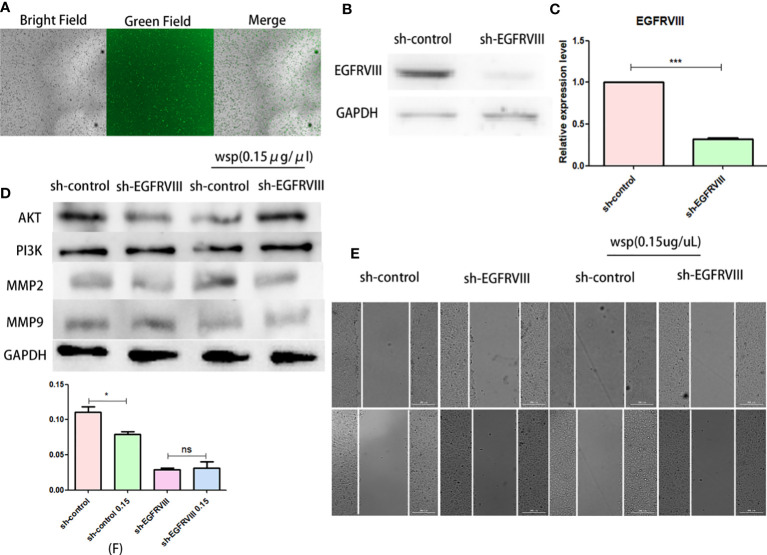
Effects of WSP on SGC-7901 migration after EGFRVIII knockdown. **(A)** SGC-7901 cells under bright field, green fluorescence field and merge after 48h siRNA transfection. **(B)** Western blotting analysis of EGFRVIII in SGC-7901 and sh-SGC-7901 after siRNA–EGFRVIII transfection. **(C)** qPCR analysis of EGFRVIII in SGC-7901 and sh-SGC-7901 after siRNA EGFRVIII transfection. **(D)** Western blotting analysis of AKT, PI3K, MMP2, and MMP9 in SGC-7901 or sh-SGC-7901 treated without or with WSP. **(E)** Qualitative of cancer number migration in SGC-7901 and sh-SGC-7901 cells treated without or with WSP as shown in **(E)**. **(F)** Quantification of cell number migration in SGC-7901 and sh-SGC-7901 cells treated without or with WSP as shown in **(F)**. *P < 0.05, ***P < 0.005, ns, no statistical difference.

To verify the role of EGFRVIII in the effect of WSP migration, we used siRNA to knock down EGFRVIII protein in SGC-7901 cells. Fluorescence microscopy (**Figure 5A**), Western blot (**Figure 5B**), and qPCR (**Figure 5C**) were used to verify cell transfection and knockout effects respectively. At 48 h after transfection, strong green fluorescence was observed under the fluorescence microscope. At 72 h qPCR and Western results showed that the expression level of EGFRVIII in cells was significantly lower than that in the untreated group. After that, EGFRVIII knockdown cells were collected for Wound-Healing Assay, and the results showed that after EGFRVIII knockdown, the expression of related proteins AKT and PI3K was reversed (**Figure 5D**) and the anti-gastric cancer migration ability of WSP was decreased (**Figures 5E, F**). It demonstrates that WSP inhibits the downstream AKT signaling pathway through EGFRVIII.

### Effects of WSP on gastric and gastric cancer organoid

**Figure 6:**
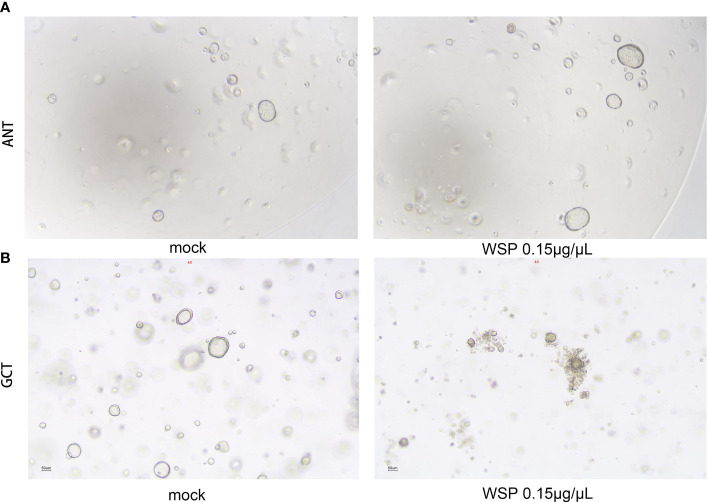
Effects of WSP on the morphology of gastric and gastric cancer organoids. **(A)** 0.15 μg/μL WSP was used to treat gastric organoid to compare the morphological changes of gastric organoids treated without and with WSP. There is no obvious change in the morphological structure of gastric organoids under the microscope. Bar: 50uM. (ANT, Adjacent Normal Tissue). **(B)** 0.15 μg/μL WSP was used to treat GC organoid, to compare the morphological changes of gastric cancer organoids treated without and with WSP. the microscopic gastric cancer organoid transform into a multilayer or irregular solid globular Bar: 50uM. (GCT, Gastric Cancer Tissue).

In view of the advantages of high fitting degree, short culture period, and stable passage of organoids, the gastric and gastric cancer environment can be accurately simulated. In order to better simulate the biological activity of WSP and understand the drug sensitivity of normal and cancer cells, we established a gastric and gastric cancer organoid model to explore the effect of WSP. After digestion, culture, and passage for at least 3 generations according to the above experimental method, the gastric and gastric cancer organoids appeared in a 3D matrix in the form of spherical cysts after microscopic examination, and the modeling was determined to be successful. Then, 0.15 μg/μL WSP was used to treat adjacent normal tissue (ANT) and gastric cancer tissue (GCT) organoids. Six days later, we found that there was no obvious change in the morphological structure of ANT organoids under the microscope (**Figure 6A**), while GCT organoids in the WSP treated group can be seen to form multi-layered or irregular solid spheres from spherical (**Figure 6B**). The GCT organoid morphology changes, while the ANT group still maintains its original spheres, tipping WSP can affect the morphology characterization of GCT organoid, and inhibit the derivative and differentiation of GCT organoid.

### WSP inhibits metastasis of gastric cancer *in vivo*

**Figure 7:**
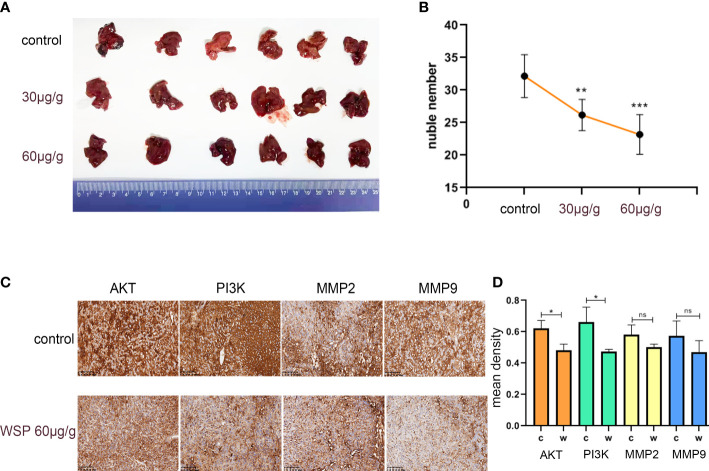
WSP inhibits liver metastasis of gastric cancer in mice. **(A)** Representative images showing the metastasis of liver cancer from gastric cancer in mice with WSP (30μg/g), WSP (60μg/g) treatment or without WSP (n=6). **(B)** Liver metastasis of mice in the control group, WSP (30μg/g) group and WSP (60μg/g) group. **(C)** IHC analysis of AKT, PI3K, MMP2, and MMP9 in cancers derived from mouse liver metastases. Bar: 20uM. **(D)** Quantification of AKT, PI3K, MMP2, and MMP9 in cancers derived from mouse liver metastases as shown in **(C)**. *P<0.05, **P<0.01, ***P <0.005 ns, no statistical difference.

Gastric cancer cells were intraperitoneally injected into mice to establish a gastric cancer metastasis model. Mice in the experimental group were intraperitoneally injected with WSP, and mice in the control group were treated with normal saline. Experimental results showed that only slight metastasis was found in mice injected with WSP, and the number of cancer nests formed in the liver was significantly lower than that in the control group (**Figures 7A, B**), suggesting that WSP significantly weakened the ability of gastric cancer cells to induce metastasis. IHC analysis was then performed on themetastatic lesions, and the results showed that the expressions of AKT and PI3K in the experimental group were significantly reduced compared with those in the control group (**Figures 7C, D**).

## Discussion

Gastric cancer is one of the most common malignant cancers of the digestive tract and the mortality rate of GC being the second highest among all malignant cancers. Therefore, it is crucial to find substances that can inhibit the signaling pathways associated with cancer migration.

**Figure 8:**
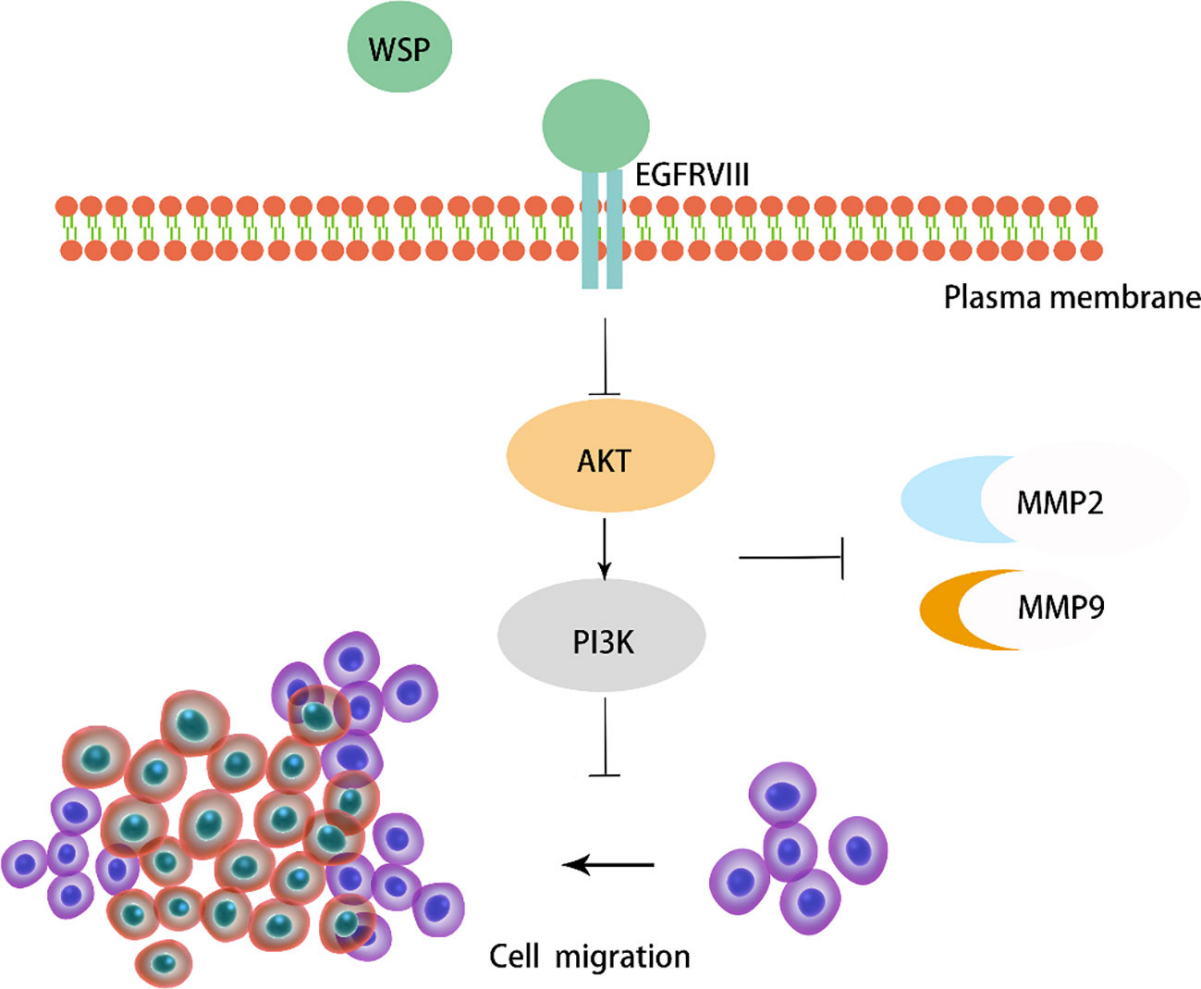
Diagram of the mechanism of action of WSP.

Previous studies have shown that WSP from “Nostoc commune” Vauch can inhibit cancer cell migration in colon cancer by affecting the downstream PI3K-AKT-mTOR signaling pathway, with little effect on normal cells (7). In our study, we validated the role of WSP in gastric cancer metastasis at the cellular level, the organoid level and in mouse models. We found that WSP can inhibit the downstream EGFRVIII signaling pathway Akt-PI3K and further inhibit the secretion of cancer-related metastasis proteins (**Figure 8**), similar to the findings of GUO S et al.

EGFR over-activation is observed in a vast number of cancers, so, targeting EGFR and its downstream signaling cascades are regarded as a rational and valuable approach in cancer therapy. The occurrence of cancer metastasis depends on cancer cell characteristics, host characteristics and their interactions. The unique ability of cancer cells to metastasize is one of the most important determinants of successful metastasis (19). Recent studies have shown that various biological characteristics of cancer cells may be closely related to the malignant behavior and metastatic ability of cancer cells (20). Therefore, WSP may affect some signal pathways that are present in gastric cancer cells but not in normal cells, thereby inhibiting the migration of cancer cells without affecting normal cells.

It is well known that cancer metastasis is closely related to various factors such as fibroblasts, transforming growth factors, chemokines and their receptors, hypoxic environment in the cancer microenvironment, so this may be the reason why the results of three cell lines are different responses to WSP migration inhibition. Although previous studies have only achieved the highest concentration of 0.1 μg/μl, and it has a good effect in colon cancer. But our study shows that WSP is dose-dependent, and the dose in mice can reach 60μg/g, and the expression of proteins related to cancer metastasis, such as MMP2 and MMP9, is observed to decrease. However, the upper limit may not be reached, and we will further conduct higher doses of WSP phenotype and toxicity experiments on more gastric cancer cells and models.

The limitations of our study are that only the EGFR-PI3K-Akt signaling pathway is performed in gastric cancer, and WSP may affect the metastasis of gastric cancer cells through other pathways. In addition, we used only one mouse model of cancer cell metastasis to verify the phenotype and effect of WSP at the animal level, which needs further study.

Given the anticancer properties of WSP, drug developers and manufacturers can further develop protein drugs for cancer patients using protein engineering techniques based on the properties of WSP. WSP can be prepared in large quantities by genetic engineering. However, for protein drugs, their molecular weight is large, and the administration cycle is long and frequent (21). How to use new biotechnology to improve the bioavailability of WSP, how to modify WSP (22, 23), and develop its stable biological activity are still worthy to be further studied.

## Data availability statement

The original contributions presented in the study are included in the article/supplementary material, further inquiries can be directed to the corresponding authors.

## Ethics statement

The studies involving human participants were reviewed and approved by Shanxi Province Cancer Hospital Ethics Committee. The patients/participants provided their written informed consent to participate in this study. The animal study was reviewed and approved by Institutional Animal Care and Use Committee of China Institute for Radiation Protection.

## Author contributions

XC, JM and WB contributed to the study conception; ZYL and JL performed most cellular and in vitro experiments with input from RS; XY, CL, ZW, LH and ZL contributed to the study conception; LS and TG worked on the visualization of the manuscript; JZ reviewed and edited the manuscript; HZ and XQL supervised the project. All authors contributed to manuscript revision, read, and approved the submitted version.
